# Analysis of Gluten in Dried Yeast and Yeast-Containing Products

**DOI:** 10.3390/foods9121790

**Published:** 2020-12-02

**Authors:** Laura K. Allred, Mitchell G. Nye-Wood, Michelle L. Colgrave

**Affiliations:** 1 Gluten Intolerance Group of North America, Auburn, WA 98092, USA; 2 School of Science, Edith Cowan University, Joondalup, WA 6027, Australia; m.nyewood@ecu.edu.au (M.G.N.-W.); michelle.colgrave@csiro.au (M.L.C.)

**Keywords:** gluten, yeast, LC–MS, ELISA

## Abstract

Yeast are commonly used in the preparation of foods and beverages such as beer and bread and may also be used on their own as a source of nutrients and flavoring. Because of the historical connection of yeast to products made from wheat and barley, consumers maintaining a gluten-free diet can have concerns about the safety of yeast ingredients. Analyzing the safety of yeast and yeast-containing products presents some difficulties, as the yeast organisms actively degrade any gluten in the product, raising questions on the appropriateness of detection by traditional antibody-based methods. This study examines a variety of yeast and yeast-containing products by competitive ELISA and liquid chromatography-mass spectrometry for the estimated level of gluten proteins. While samples such as yeast extracts and nutritional yeast contained gluten levels below the 20 mg/kg (or parts per million, ppm) threshold defined by Codex Alimentarius, one baking yeast and a nutritional yeast supplement sample contained higher levels of gluten. This study demonstrates that both competitive ELISA and liquid chromatography-mass spectrometry provide similar results in the detection of wheat and barley gluten in yeast-containing products.

## 1. Introduction

While yeast have been used commercially for fermentation for thousands of years, only in the past 150 years have manufacturers harnessed specific yeast strains for their innate nutritional value and sensory properties [1,2,3]. Yeast are essential for fermentation and leavening in such staples as bread, baked goods, beer, and wine, and are used as protein and flavoring sources in the food supply [4]. Given their links to products typically made with grains such as wheat and barley, consumers with celiac disease (CD) and gluten sensitivity can harbor concerns about the gluten-free status of yeast, and the foods and beverages made with them. Accurate test methods are essential for determining the gluten content of these products.

Yeast are single-celled fungi. As a protein source, they are much easier and less expensive to cultivate and harvest than crops or livestock, because they can be grown on inexpensive by-product sources [5,6], such as molasses from the sugar industry [7]. Like many other microorganisms, they have a rapid growth rate and a high protein content [4].

Most commercial yeast are strains of the genus Saccharomyces [1]. Yeast are commonly referred to by their intended function, for example as baker’s yeast, brewer’s yeast, nutritional yeast, distiller’s yeast, wine yeast, or probiotic yeast. Torula yeast is a different yeast strain, genus Candida, grown in molasses or cellulose (wood) waste and used as a nutritional supplement for humans and animals [3]. 

Because Saccharomyces yeast are used across multiple industries and are often grown on wheat or barley substrates when used for processes such as beer fermentation or bread leavening, persons who avoid gluten due to celiac disease or other gluten intolerances are often unsure about the safety of products that include yeast or yeast extracts. Adding to this confusion is the use of common names for yeast preparations that may not accurately indicate how they are grown. Brewer’s yeast used in the beer industry may be yeast that have been collected from a previous batch of beer [8], but small-batch brewers are often using dried yeast that was grown on sugar cane or beet molasses [9,10]. The dead “spent” yeast from the beer industry may be used to make brewer’s yeast extracts like the traditional Marmite [11] and Vegemite spreads [12], which can contain high levels of residual gluten [13]. However, many powdered yeast extracts used as flavoring sources are grown on cane sugar molasses, beet molasses or other non-gluten sources [14,15,16]. Baker’s yeast can similarly be purchased as a dried, industrially processed product that has never been grown on wheat [17,18], while a sourdough starter will typically contain wheat flour and high levels of gluten.

The purpose of the current analysis is to preliminarily examine the capabilities of LC–MS for the detection and quantitation of gluten in yeast, through comparison to an existing validated method. Along with industrial and retail samples of dried yeast used as a flavoring component, samples of baker’s yeast and brewer’s yeast from various sources were analyzed.

## 2. Materials and Methods

Twenty samples were collected from industrial and retail sources. The samples were either pure dried yeast preparations, or gluten-free products containing yeast extract as an ingredient. None of the materials tested made a gluten-free claim. The sample identities are shown in Table 1.

Because yeast are living organisms, any gluten or other proteins in their growth media have the potential to be broken down by the yeast’s enzymes—effectively making the growth media similar to a fermented product. This fermentation process makes traditional sandwich ELISA test methods unsuitable for detecting and quantitating gluten [19]. Therefore, the competitive antibody-based ELISA method was used to analyze the samples for gluten content. Subsequently, analysis for the presence of gluten was performed by liquid chromatography–tandem mass spectrometry (LC–MS/MS). This method provided the added benefit of being able to speciate any gluten residues as having come from wheat or barley.

Competitive ELISA. Samples were analyzed by RIDASCREEN^®^ gliadin competitive ELISA (R7021, R-Biopharm, Sydney, Australia) according to the manufacturer’s instructions. All analyses were conducted in duplicate and the plates were read using a Varioskan LUX multimode microplate reader (Thermo Scientific, Scoresby, Australia). A cubic spline function was fit to the absorbance of the gliadin standards in RIDASOFT^®^ Win.NET software (R-Biopharm, Sydney, Australia), and used to interpolate the unknown values. These values were multiplied by the dilution factor of 500 to yield the concentration of gliadin in mg/kg. To convert gliadin into gluten, a multiplication factor of 2 was used as recommended by the Codex Alimentarius. 

Sample preparation for LC–MS analysis. The samples were weighed out in quadruplicate, and gluten was extracted for proteomic analysis as previously described [20]. The 20 experimental yeast samples were prepared and later analyzed alongside two positive controls of mixed wheat and barley flour. These controls comprised a range of commercial cereal cultivars to ensure that all gluten classes were represented. The wheat positive control consisted of equal parts of wheat cultivars Alsen, Xiayan, Pastor, Westonia, Baxter, Chara, Yitpi, AC Barrie, and Volcania. The barley positive control consisted of equal parts of barley cultivars Flagship, Hindmarsh, Sloop, Oxford, Baudin, Yagan, Bomi, Fleet, Commander, Gairdner, Scope, and Maritime. Yeast samples of approximately 20 mg were extracted using 55% IPA/2% DTT solution (10:1 *w*/*v*, approximately 200 µL) for 30 min at 50 °C. Four replicates were prepared per sample (labelled A-D in figures below). The supernatant (100 µL) after centrifugation (20,800 *g*, 15 min) was applied to a 10 kDa molecular weight cut-off filter and washed twice with a buffer consisting of 8 M urea in 0.1 M Tris-HCl (pH 8.5). The proteins were alkylated by incubation with 50 mM iodoacetamide for 20 min at RT in the dark. Buffer exchange using 100 mM ammonium bicarbonate (pH 8.5) was undertaken by two consecutive wash/centrifugation steps (20,800× *g*, 10 min) before the application of trypsin (Promega, Alexandria, Australia), at 20:1 protein:enzyme *w*/*w*) in 100 ammonium bicarbonate, 1 mM CaCl_2_ (pH 8.5) and 16 h incubation at 37 °C in a wet chamber. The filters were transferred to fresh centrifuge tubes and the filtrates (digested peptides) were collected following centrifugation (20,800× *g*, 10 min). The filters were washed with 200 μL of 100 mM ammonium bicarbonate and the filtrates were combined and lyophilized. The tryptic peptides were resuspended in 100 μL of 1% formic acid and stored at 4**** °C until analysis. 

Targeted MS. Reduced and alkylated tryptic peptides were chromatographically separated on an Exion AD UHPLC system (SCIEX, Redwood City, CA, USA), and analyzed on a 6500+ QTRAP mass spectrometer (SCIEX). Data acquisition was achieved using scheduled MRM scanning experiments using a 60 s detection window for each MRM transition and a 0.3 s cycle time. Three transitions were monitored per peptide, and two peptides per protein. Peaks were integrated using Skyline [21] wherein all three transitions were required to co-elute at the same retention time (RT) with a signal-to-noise (S/N) > 5. Graphs were exported from Skyline or generated in Graphpad Prism v8. 

Following analysis of a 1 µL injection volume, analyses were repeated with a higher range of injection volumes (1, 2, and 5 µL) to look for increased peak area and confirm detection. Finally, the concentration of gluten in the samples were estimated by comparing the observed gluten peptide signal abundance to that of positive control wheat or barley samples diluted in a solution of gluten-free yeast extract. 

Comments on estimation. Due to the unknown levels of gluten hydrolysis in each sample, precise quantitation of gluten was not possible. Absolute quantitation would require further analysis using incurred yeast samples (that were found to be gluten-free) with the same wheat (or barley) cultivar used in yeast propagation for subsequent analysis. The estimates shown in the LC–MS/MS graphs are based on relative quantitation by comparison to the gluten levels observed in positive controls of wheat and barley samples. The first assumption is made based on the average protein level of wheat/barley of 12% (the range can be ~9–14%). The second assumption is that gluten comprises ~75% of wheat protein and ~50% of barley protein [22]. Deviations from these assumed values would cause the estimate to be inaccurate. These estimates are made only to give an indication of whether the trace levels are at, or above, the Codex Alimentarius threshold of 20 mg/kg (ppm).

## 3. Results

The yeast samples were analyzed by two methods in parallel—the traditional method employing competitive ELISA and the more advanced LC–MS/MS methodology. Using ELISA (Table 1), gluten was detected at very high levels in samples 18 and 20, wherein the levels were well above the upper limit of quantitation of 270 mg/kg. Sample 17 also reflected a moderate level of gluten at ~16 mg/kg, close to the threshold of 20 mg/kg. All other samples yielded gluten levels below the 10 mg/kg lower limit of quantitation. The samples were also analyzed using two LC–MS/MS methods that focused on detection of wheat and barley. The highest levels were also noted in samples 18 and 20 correlating with the results of the ELISA. Likewise, sample 17 also showed low levels of wheat and barley. In addition, trace levels of wheat were detected in samples 1, 12, 14, and 19; and of barley in sample 8.

**Figure 1:**
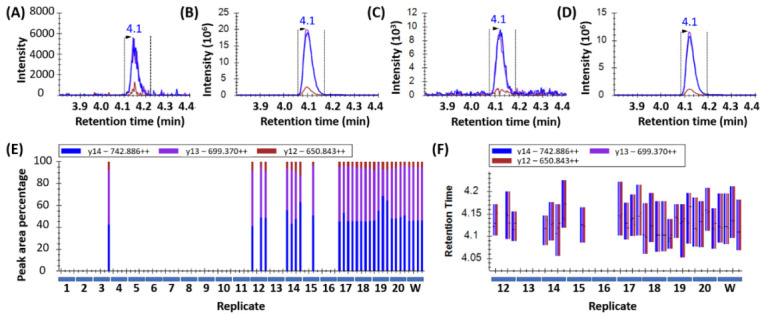
Wheat peptide (DVSPGCRPITVSPGTR from HMW-GS, Uniprot: Q45R38) peak detection in yeast samples: (**A**) Sample 17 (intensity 6.0 × 10^3^); (**B**) sample 18 (intensity 2.0 × 10^7^); (**C**) sample 20 (intensity 1.0 × 10^4^); and (**D**) wheat (control, intensity 1.2 × 10^7^). The peaks were detected using three MRM transitions per peptide and the ratio of these as shown in (**E**) by blue/purple/brown bars act as a quality control (QC) factor for peak detection. A second QC factor was the co-elution of the transitions at the retention time of the control wheat (**F**).

Peak detection in wheat samples is displayed in Figure 1. Peptides were considered confidently detected when all three transitions per peptide co-eluted at the correct retention time with the same pattern (order of intensity) and an intensity >1000 counts per second (cps). This is depicted in Figure 1A for sample 17 which showed low but detectable levels of wheat, as well as in samples 18, 20, and the wheat control in Figure 1B, Figure 1C, and Figure 1D respectively. The consistent ratio of abundance of the transitions are shown in Figure 1E, and the consistent retention time is shown in Figure 1F. 

**Figure 2:**
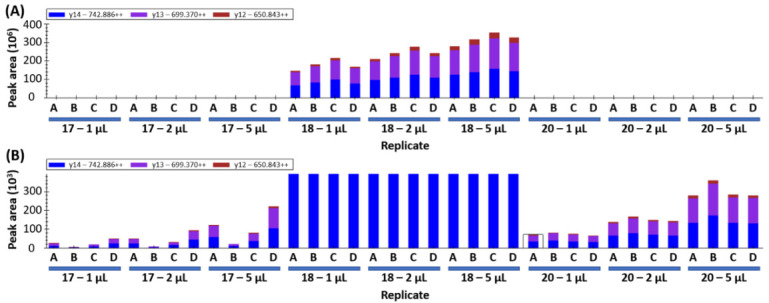
Wheat peptide (DVSPGCRPITVSPGTR from HMW-GS, Uniprot: Q45R38) peak area measurement using increasing injection volumes (1, 2, and 5 µL) of samples 17, 18, and 20. The four technical replicates are indicated by the letters A–D, the colored columns indicate the peak area for the three MRM transitions. (**A**) shows the full range of peak area, whilst (**B**) shows a zoomed in *y*-axis allowing the view of the trace level gluten detection in samples 17 and 20.

If the peptides detected in yeast samples are wheat-specific gluten peptides, increasing the injection volume would be expected to cause a proportional increase in peak area. This was assessed by repeating data collection with an injection volume of 1 µL, 2 µL, and 5 µL on samples 17, 18, and 20. A representative peptide is shown in Figure 2, where the increase in peak area for sample 18 is evident at larger injection volumes (Figure 2A). The increase is less than the expected ratios of 2 and 5, likely because the MS detector is being saturated by the hyperabundance of this peptide in the sample. Yeast samples 17 and 20 show the expected 2x and 5x increase in peak area as injection volumes are increased (Figure 2B). The data presented for sample 20 shows increasing peak area that matches the increased volume and good consistency between biological replicates (18% co-efficient of variation, CV). 

The variation in abundance between technical replicates A–D is noted wherein sample 17D yields a higher peak area and 17B shows the lowest peak area (Figure 2B). The variation is attributed to “hot spots” within the inhomogeneous yeast sample as opposed to technical error during sample preparation. Similar trends can be observed in samples 18 and 20 (Figure 2). This variability indicates that higher sampling numbers and/or larger sampling amounts will be required to account for the sample inhomogeneity.

**Figure 3:**
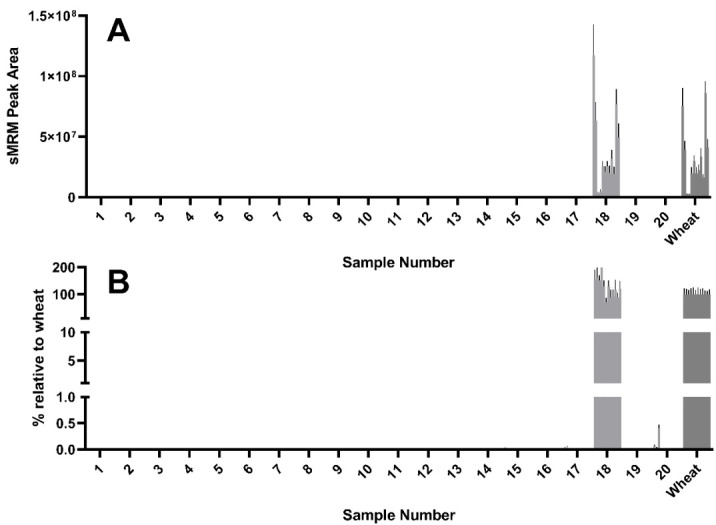
Detection of wheat marker peptides by LC–MS. Significant levels of wheat were detected in samples 17, 18, and 20, with trace levels in samples 1, 3, 5, 12, 14, 15, and 19. (**A**) LC–MS/MS peak area of the 12 wheat peptides used as peptide markers for wheat (raw data). (**B**) Peak area normalized to wheat control, expressed as a percentage.

Of the 12 wheat peptides used to detect and quantify wheat experimental samples, all 12 were detected in sample 18, a dried sourdough starter culture. Curiously, the abundance of these peptides as reflected by the peak area were comparable to, and in some cases higher than, that seen in the mixed wheat control (Figure 3A). Some but not all of the 12 wheat peptides were detected in yeasts #17 and #20, though this was more evident after normalizing to the abundance seen in the wheat control (Figure 3B). 

**Figure 4:**
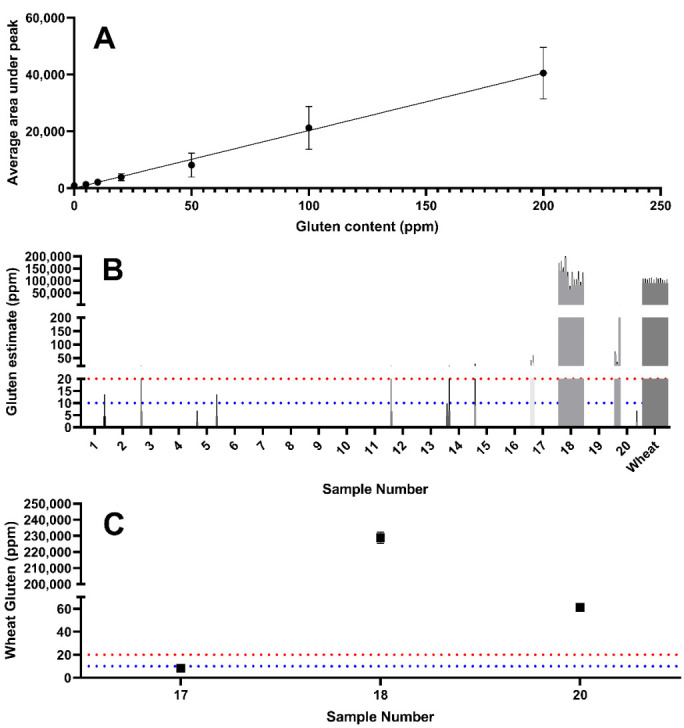
Estimates of the concentration of gluten in the samples were made by comparing to wheat samples diluted in a solution of gluten-free yeast extract. Samples were weighed independently in quadruplicate and peptide peak area measured and plotted as a calibration curve (**A**). This data was then used to generate estimates of gluten concentration across all tested samples (**B**) with clear detection in samples 17, 18, and 20 for 12 peptides (*n* = 4 replicates). Wheat gluten estimate based on the sample mean of all wheat gluten peptides and interpolated from the calibration curve (**C**). The dashed red line represents the 20 mg/kg threshold, whilst the dashed blue line represents the 10 mg/kg GFCO certification threshold.

Using a combination of yeast extracts that were observed to be wheat-free (i.e., samples 2, 7, 8, and 16), wheat was spiked in at a known concentration to generate a yeast extract solution with a composition comparable to that of the unknown samples. This accounted for matrix effects and enabled the generation of a standard curve to enable estimation of the wheat gluten content (Figure 4A). The standard curve was used to estimate the gluten content using the peak area determined by LC–MS/MS. 

While wheat derived gluten was detectable in sample 17, the levels are likely to be below a threshold of 20 mg/kg correlating with the ELISA determination of 16.1 mg/kg (Table 1). Sample 18 showed extremely high intensity wheat peptide peak areas (228,910 mg/kg), which were higher than those observed in the untreated wheat positive control. This may suggest that wheat peptides are enriched during the manufacturing process of this product. Sample 20 also revealed a wheat gluten estimate above 20 mg/kg.

**Figure 5:**
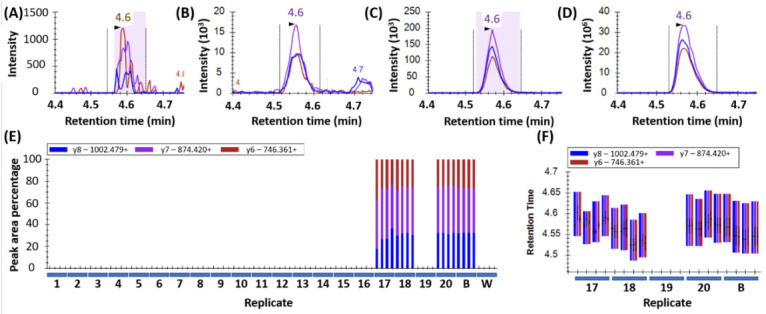
Barley peptide (VFLQQQCSPVR from B-hordein, UniProt: I6SJ22) peak detection in yeast samples: (**A**) sample 17 (intensity 1.2 × 10^3^); (**B**) sample 18 (intensity 1.7 × 10^4^); (**C**) sample 20 (intensity 2.0 × 10^4^); and (**D**) barley (control, intensity 3.4 × 10^7^). The peaks were detected using three MRM transitions per peptide and the ratio of these as shown in (**E**) by blue/purple/brown bars act as a quality control (QC) factor for peak detection. A second QC factor was the co-elution of the transitions at the retention time of the control barley (**F**).

Peak detection in barley samples is displayed in Figure 5. As with the wheat detection experiments, barley peptides were considered confidently detected when all three transitions per peptide co-eluted at the correct retention time with the same pattern (order of intensity) and an intensity >1000 counts per second (cps). This is shown in Figure 5A for sample 17 which showed low but detectable levels of barley, as well as in samples 18, 20 and the mixed barley control in Figure 5B, Figure 5C, and Figure 5D respectively. The consistent ratio of the MRM transition peak areas is shown in Figure 5E, and the consistent retention time is shown in Figure 5F. 

**Figure 6:**
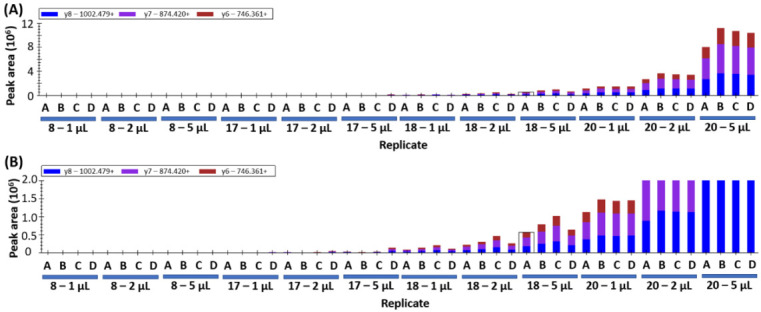
Barley peptide (VFLQQQCSPVR from B-hordein, UniProt: I6SJ22) peak area measurement using increasing injection volumes (1, 2, and 5 µL). The four technical replicates are indicated by the letters A-D, the colored columns indicate the peak area for the three MRM transitions. (**A**) shows the full range of peak area, whilst (**B**) shows a zoomed in *y*-axis allowing the view of the trace level gluten detection in samples 17 and 20.

Increasing the injection volume from 1 µL to 2 µL and 5 µL for samples 8, 17, 18, and 20 revealed an increase in peak area which is evident for sample 20 (Figure 6A). The data presented for sample 20 shows increasing peak area that matches the increased volume and good consistency between biological replicates (overall CV 11%). Trace barley levels observed in some replicates of yeast sample 8 were barely detectable (Figure 6B), whilst yeast samples 17 and 18 showed robust detection of low levels of barley gluten that increased with increasing injection volume (Figure 6B). As was observed for the wheat experiments above, variation in abundance between technical replicates was consistent across injections, suggesting that the variation is due to “hot spots” such as in sample 18C due to the inhomogeneous nature of the yeast samples (Figure 6A,B). 

**Figure 7:**
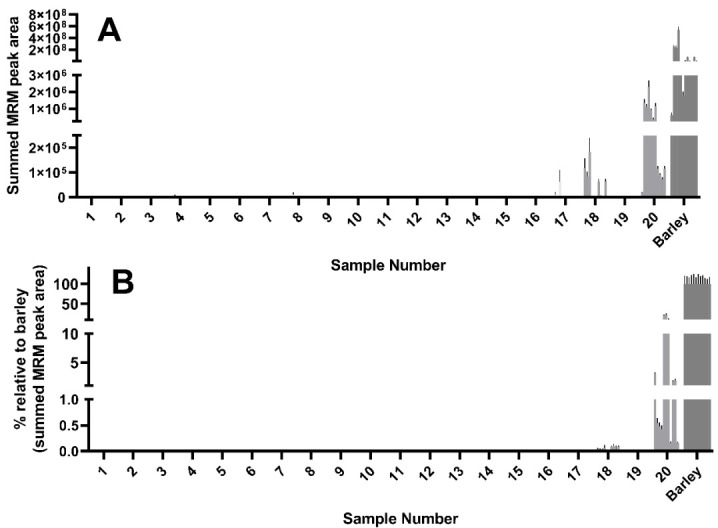
Detection of barley marker peptides by LC–MS. Significant levels of barley were detected in samples 17, 18 and 20, with trace levels detected in samples 8 and 14. (**A**) LC–MS peak area of 9 barley peptide markers (raw data). (**B**) Peak area normalized to barley expressed as a percentage.

Of the 12 barley peptides used to detect and quantify barley in experimental samples, all 12 were detected in sample 20. Sample 18, which showed high levels of wheat gluten content, showed some but not all barley specific peptides (Figure 7A,B). Yeast samples 4, 8, and 17 showed low levels of some but not all barley peptides (Figure 7A,B). 

**Figure 8:**
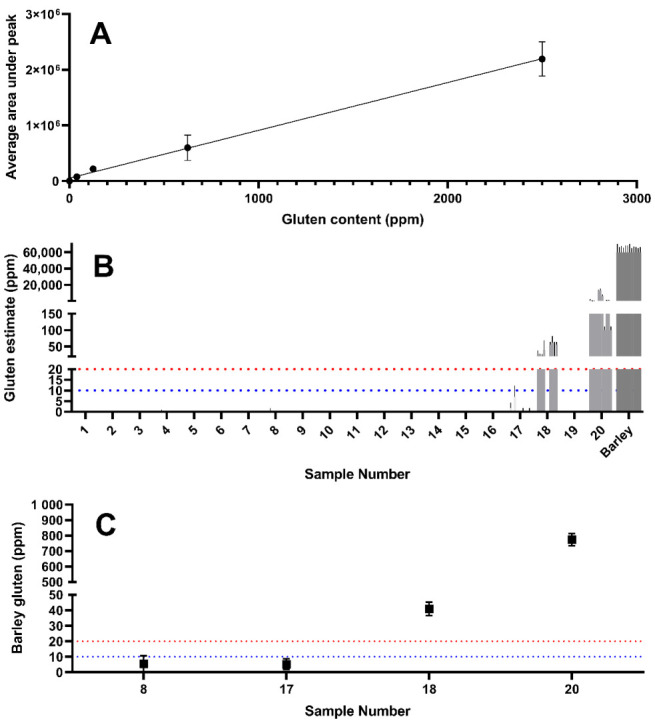
(**A**) Standard curve of barley gluten in yeast extract. Barley-derived gluten was spiked into gluten-free yeast extract at known concentration. (**B**) Estimates of barley-derived gluten concentration in samples 8, 17, 18, and 20 (*n* = 4 replicates). (**C**) Barley gluten estimate based on the sample mean of all barley gluten peptides and interpolated from the calibration curve. The dashed red line represents the 20 mg/kg threshold, whilst the dashed blue line represents the 10 mg/kg GFCO certification threshold.

Barley gluten was estimated in yeast samples using the same approach as described above for wheat. A combination of barley-free yeast extracts (samples 3, 5, 7, and 10) was generated into which barley was spiked at a known concentration in order to negate any matrix effects and enable a standard curve to be generated with which barley gluten content could be estimated (Figure 8A). The barley gluten content was estimated as shown in Figure 8C using this standard curve and the peak areas displayed in Figure 7A. This approach estimates that the barley gluten detected in samples 8 and 17 were likely to be below the 20 mg/kg regulatory threshold defined by the Codex Alimentarius, while barley gluten in samples 18 (~41 mg/kg barley) and 20 (~772 mg/kg barley) is estimated to be significantly higher than 20 mg/kg. 

## 4. Discussion

Two of the samples analyzed, a sourdough starter culture (Sample 18) and a brewer’s yeast nutritional supplement (Sample 20), contained wheat and barley gluten at levels estimated to be greater than 20 ppm by both the competitive ELISA and LC–MS/MS methods. 

Sourdough is leavened using the naturally occurring yeast and bacteria present in flour. Although claims have been made regarding the safety of sourdough products for persons with celiac disease [23], traditional sourdough leavening alone does not remove gluten from wheat-based products, but it can improve the textural and structural properties of products made from gluten-free flours [24]. The data presented here indicate the need for gluten-free home bakers and manufacturers alike to ensure that they are starting with a sourdough culture that has been grown on gluten-free flour. While high levels of wheat gluten were expected for this sample, the results indicated that the sample was concentrated such that the peak areas were equivalent to the wheat control. Moreover, LC–MS/MS analysis also identified high levels of barley, estimated ~40 mg/kg.

Brewer’s yeast supplements are often spent yeast from the brewing industry, so the high barley gluten level (~772 mg/kg) in this sample was not unexpected. The sample also contained wheat gluten at an estimated level of 61 mg/kg as measured by LC–MS/MS. Agricultural co-mingling of grains is not uncommon, and could account for these results, as could the prior use of the brewer’s yeast in the preparation of wheat-based beers.

None of the six yeast extracts, or products containing yeast extract (Samples 1–11), were found to contain gluten at a level above 20 mg/kg. However, three of these samples had a wheat gluten level that was estimated to be at or near the 10 mg/kg threshold used by the Gluten Free Certification Organization (GFCO) when analyzed by LC–MS/MS. All of the yeast extracts and products containing yeast extract tested at less than 10 mg/kg using the competitive ELISA. The primary concern with yeast extracts for consumers that are gluten-free is the possibility that they may have been sourced from the beer brewing industry, but no barley gluten levels greater than 10 ppm were detected by LC–MS/MS in the yeast extracts or products containing yeast extract. While this was not an exhaustive survey of yeast extracts used as flavoring components, these results from multiple random samples provide an indication that powdered yeast extracts used as flavoring are often grown on gluten-free substrates. It is important to remember that dried yeast extracts are not typically sold in their concentrated form to consumers and are used at very low levels in finished products. This contrasts with paste yeast extracts, such as Marmite and Vegemite, that may be made from spent yeast obtained as a by-product from the brewing industry and sold directly to consumers [11,12]. 

The two nutritional yeast samples that were tested did not have estimated gluten levels above 10 mg/kg by either competitive ELISA or LC–MS/MS. Nutritional yeast are grown similarly to those used to prepare yeast extracts, but while yeast extracts consist of only the soluble fraction of autolyzed yeast, nutritional yeast is a dried preparation of the whole yeast, including the soluble and insoluble fractions.

Gluten-free consumers should be aware of the difference between nutritional yeast and brewer’s yeast nutritional supplements. In this small sample set, the nutritional yeast products did not contain gluten at a level above the Codex or GFCO thresholds, while the brewer’s yeast powder contained high levels of barley and wheat gluten, indicating likely preparation from spent yeast from the brewing industry. In contrast, Sample 19, a Dry Ale Yeast sold for home beer production, did not contain detectable barley gluten contamination, and only trace levels of wheat gluten.

While samples 14–17 were all dried baker’s yeast products, samples 14–16 were commercial brands, commonly sold for consumer use. Sample 17 was in non-commercial packaging, locally sourced by a health food store. 

The quantitative LC–MS/MS data provided in this study are estimated, but these results indicate that residual gluten in yeast can be detected, and the specific grain source can be determined, by LC–MS/MS. This work also indicates that the competitive ELISA and LC–MS/MS produce similar results for yeasts and yeast extracts. Further studies using incurred samples derived from the same source materials (wheat or barley cultivars) would help to determine the ability to accurately quantitate gluten in yeast samples.

## Figures and Tables

**Table 1:** Sample identities and relative wheat and barley gluten content.

Sample	Sample Description	LC–MS Wheat Level	LC–MS Barley Level	ELISA (mg/kg)
1	Dried yeast extract, used as flavoring	+	-	<10
2	Dried yeast extract, used as flavoring	-	-	<10
3	Dried yeast extract, used as flavoring	-	-	<10
4	Dried yeast extract, used as flavoring	-	-	<10
5	Dried yeast extract, used as flavoring	-	-	<10
6	Dried yeast extract, used as flavoring	-	-	<10
7	Seasoning blend with yeast extract	-	-	<10
8	Seasoning blend with yeast extract	-	+	<10
9	Seasoning blend with yeast extract	-	-	<10
10	Seasoning blend with yeast extract	-	-	<10
11	Snack product with yeast extract as flavoring	-	-	<10
12	Nutritional yeast flakes	+	-	<10
13	Seasoning with nutritional yeast	-	-	<10
14	Dried baker’s yeast	+	-	<10
15	Dried baker’s yeast	-	-	<10
16	Dried baker’s yeast	-	-	<10
17	Dried baker’s yeast	+	+	16.1
18	Dried sourdough starter culture	+++	++	>270
19	Dried brewer’s yeast for beer production	+	-	<10
20	Dried brewer’s yeast nutritional supplement	++	+++	>270

− = not detected, + = detected at a level estimated to be equivalent to less than 20 mg/kg gluten, ++/+++ = detected at a level estimated to be equivalent to greater than 20 mg/kg gluten.
